# The Multiple-Demand System in the Novelty of Musical Improvisation: Evidence from an MRI Study on Composers

**DOI:** 10.3389/fnins.2017.00695

**Published:** 2017-12-11

**Authors:** Jing Lu, Hua Yang, Hui He, Seun Jeon, Changyue Hou, Alan C. Evans, Dezhong Yao

**Affiliations:** ^1^The Clinical Hospital of Chengdu Brain Science Institute, MOE Key Lab for Neuroinformation, University of Electronic Science and Technology of China, Chengdu, China; ^2^Montreal Neurological Institute, McGill University, Montreal, QC, Canada; ^3^Center for Information in BioMedicine, School of Life Science and Technology, University of Electronic Science and Technology of China, Chengdu, China; ^4^Department of Composition, Sichuan Conservatory of Music, Chengdu, China

**Keywords:** functional MRI, structural MRI, musical improvisation, multiple-demand system, neuroplasticity

## Abstract

The multiple-demand (MD) system has proven to be associated with creating structured mental programs in comprehensive behaviors, but the functional mechanisms of this system have not been clarified in the musical domain. In this study, we explored the hypothesis that the MD system is involved in a comprehensive music-related behavior known as musical improvisation. Under a functional magnetic resonance imaging (fMRI) paradigm, 29 composers were recruited to improvise melodies through visual imagery tasks according to familiar and unfamiliar cues. We found that the main regions of the MD system were significantly activated during both musical improvisation conditions. However, only a greater involvement of the intraparietal sulcus (IPS) within the MD system was shown when improvising with unfamiliar cues. Our results revealed that the MD system strongly participated in musical improvisation through processing the novelty of melodies, working memory, and attention. In particular, improvising with unfamiliar cues required more musical transposition manipulations. Moreover, both functional and structural analyses indicated evidence of neuroplasticity in MD regions that could be associated with musical improvisation training. These findings can help unveil the functional mechanisms of the MD system in musical cognition, as well as improve our understanding of musical improvisation.

## Introduction

The understanding of the mechanisms of complex tasks is far from clear. A major difficulty in the investigation of complex actions is decomposing the components responsible for different aspects of a behavior (Coffey and Herholz, 2013). In addition, individuals may have differences in structural and functional properties of the brain that also affect decomposing and learning in complex tasks (Zatorre, 2013). One important finding is the multiple-demand (MD) system of the frontal and parietal cortex, which is activated during many complex cognitive activities (Duncan, 2013; Crittenden and Duncan, 2014). Musical improvisation, usually considered one of the most unexplored forms of creativity, also involves comprehensive activities of cognition (Dietrich, 2003). Because most complex tasks would necessarily relate to the MD system, we are eager to know the relationship between the MD system and musical improvisation.

The MD system consists of several areas in the prefrontal and parietal regions, including the posterior part of the inferior frontal sulcus (IFS), the anterior insula and adjacent frontal operculum (AI/FO), the presupplementary motor area and adjacent dorsal anterior cingulate (pre-SMA/ACC), and the intraparietal sulcus (IPS). Occasionally, activity can also be seen in the rostrolateral prefrontal cortex (RPFC) (Duncan, 2010; Fedorenko et al., 2011). These regions have been shown to participate in many different functions, such as response selection, working memory, and task novelty (Duncan and Owen, 2000; Cole and Schneider, 2007). In working memory, a functionally connected cognitive control network involves the frontal and parietal regions (Cabeza and Nyberg, 1997). One study has found that when the retention interval is short, the occipital, and right frontal regions are significantly activated; however, when there is a longer retention interval, the involvement of parietal and left frontal regions is prominent (Haxby et al., 1995). Regarding task novelty, the frontal lobe contributes its executive functions to the early learning stage (Duncan and Owen, 2000), which has also been proved by a lesion study (Rogers et al., 1998). Moreover, an ERP study showed that the parietal cortex could be affected during novel complex tasks after prefrontal damage (Knight and Scabini, 1998).

Music is a universal human activity involving perceptually discrete elements organized into hierarchically structured sequences (Patel, 2003). Scientists have devoted themselves to uncovering the brain mechanisms of music (Zatorre et al., 2007; Bermudez et al., 2009). Musical improvisation, which requires rich musical background memories and creative novelty competencies, can be an important model to investigate the musical brain (Gross and Seashore, 1941; Lu et al., 2015). A remarkable problem is whether the MD system is also involved in musical improvisation activities.

Until now, studies on musical improvisation mainly assessed the role of the frontal regions. A functional magnetic resonance imaging (fMRI) study found that the dorsal premotor area, the rostral cingulate region and the inferior frontal gyrus are recruited for the invention of novel motor sequences in musical improvisation (Berkowitz and Ansari, 2008). Another fMRI study showed that improvisation is consistently characterized by a dissociated pattern of activity in the prefrontal cortex (Limb and Braun, 2008). Additionally, the effect of training on improvisation is positively associated with functional connectivity of the dorsolateral prefrontal cortex and dorsal premotor cortex (Pinho et al., 2014). In addition, the activation and the functional connectivity of other areas have also been shown to be linked with musical improvisation. The perisylvian language area is related to the processing of syntactic elements in music by an interactive improvisation between two musicians (Donnay et al., 2014). The functional connectivity of the bilateral occipital lobe and bilateral postcentral cortex decreases during musical improvisation, while the functional connectivity between the anterior cingulate cortex, the right angular gyrus, and the bilateral superior frontal gyrus appears significantly stronger (Lu et al., 2015). From these studies above, we can extrapolate that only the frontal regions and a few other specific areas are suggested to be involved in musical improvisation, the question of whether the MD system modulates musical improvisation has not been discussed yet.

Here, we used fMRI to study neural activity during imagery improvisations based on two different cues. One was a familiar cue, which was mainly considered to be involve working memory. The other was an unfamiliar cue, which was thought to be highly involved in creative novelty. We recruited 29 composers who had systematic knowledge of how to conceive a novel piece of music as our participants. General linear model (GLM) analysis was conducted to investigate the neural activity involved in improvisation under different conditions (Woolrich et al., 2004; Kriegeskorte and Bandettini, 2007). We used a task to investigate whether the MD system is involved in musical improvisation. We calculated the correlation between activated regions and musical improvisational level (MIL) scores to explore how the level of musical improvisation impacted brain activity. Furthermore, to reveal plastic evidence of musical improvisation, 31 non-musicians were recruited as the control group. We compared structural covariance within the MD system between composers and non-musicians.

## Methods

### Participants

Twenty-nine composers (14 males, aged 18–23 years) selected through a musical background questionnaire from the Department of Composition at Sichuan Conservatory of Music participated in the experiment. All composers had experience playing piano, which was regarded as the fundamental skill for studying musical improvisation. They all had training in musical improvisation for at least three years. All participants passed the MIL exam, which is considered an objective assessment of improvisation level. Scores of the exam were decided by the committee consisting of ten professors from the Department of Composition at Sichuan Conservatory of Music. Thirty-one non-musicians without a musical training background from the University of Electronic Science and Technology of China were recruited as the control group. Participants were all right-handed according to the Edinburgh Inventory (Oldfield, 1971) with normal hearing and vision and no history of neurological disorders. This study was carried out in accordance with the recommendations of the Ethics Committee of the School of Life Science and Technology at University of Electronic Science and Technology of China (UESTC) with written informed consent from all subjects. All subjects gave written informed consent in accordance with the Declaration of Helsinki. The protocol was approved by the Ethics Committee of the School of Life Science and Technology at UESTC.

### Procedures

#### Task design

Firstly, we need to clarify the use of this phrase “improvisation.” It is commonly accepted that the notions of composition and improvisation in making music are almost overlapped. Thus, we adopted “improvisation” in our manuscript to be in consistent with previous studies.

**Figure 1:**
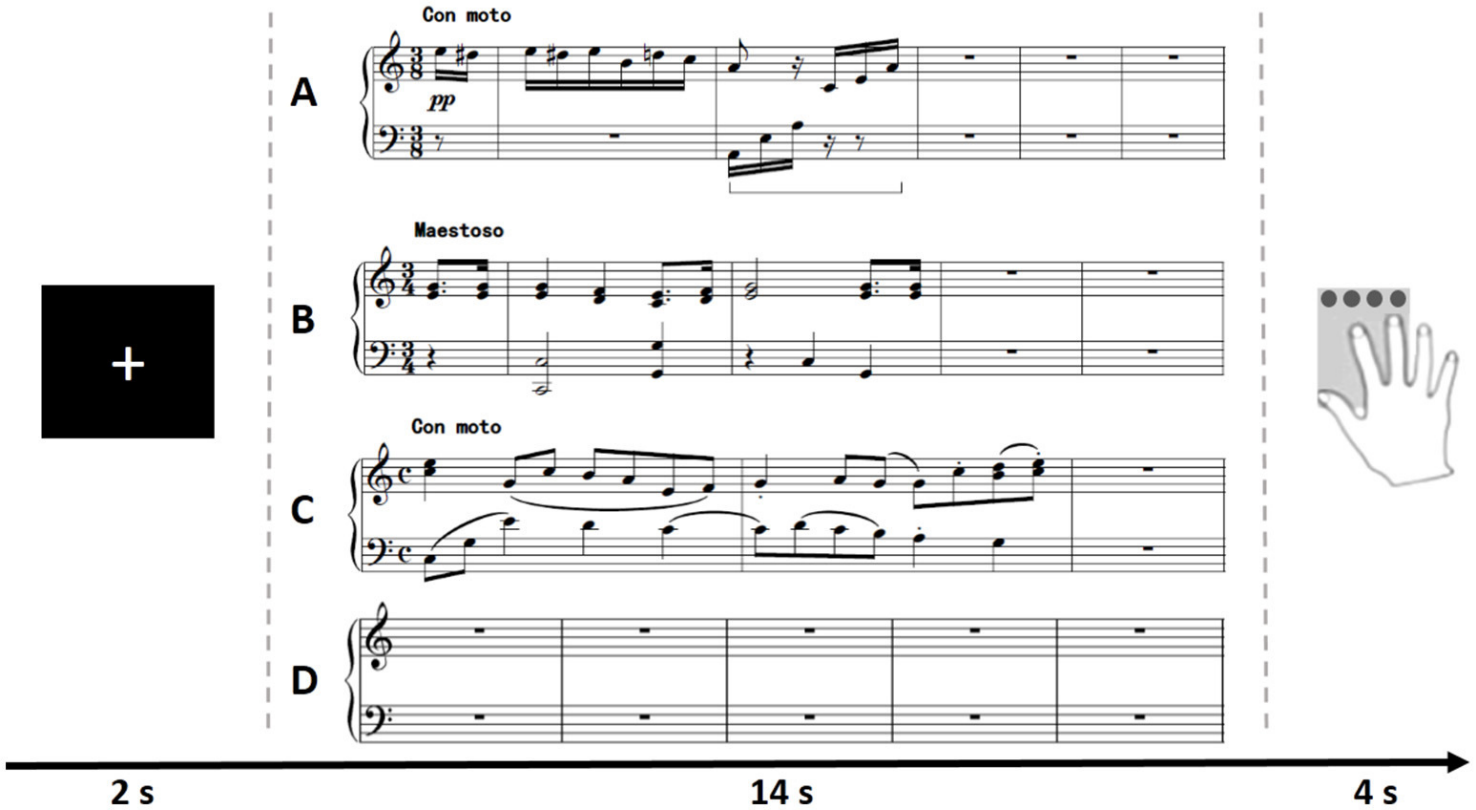
Illustration of the trials of all conditions in the experimental paradigm. For each trial, participants were asked to imagine improvising a melody piece according to different cues after a cross was shown for 2 s. The *Familiar* condition (**A**, used in both pilot and scanning), *Unfamiliar* condition (**B**, used in pilot; **C**, used in scanning) or *Baseline* condition (**D**, used in both pilot and scanning) appeared randomly and lasted for 14 s in the paradigm. After each imagery improvisation, participants were asked to give an evaluation score (from 0-unsatisfied to 3-satisfied) of their performance in a box within 4 s.

The familiar vs. unfamiliar design was widely used in musical-related research (Halpern and Zatorre, 1999; Herholz et al., 2012). Using this design, we could not only test whether MD system is involved in musical improvisation, but also find more details about improvising with these two different ways. The universally familiar melody of “Fur Elise” written by Ludwig van Beethoven was used as the familiar cue stimulus material (denoted as *Familiar*, Figure 1A). The unfamiliar cues (denoted as *Unfamiliar*) were written by a senior composer from the Sichuan Conservatory of Music so that we could ensure that none of the participants had seen these cues before. One unfamiliar cue was presented during the pilot (Figure 1B), and the other unfamiliar cue was presented during fMRI scanning (Figure 1C). A blank stave was used as the baseline condition (denoted as *Baseline*, Figure 1D).

The tasks were designed and presented with E-prime 2.0 software. For each trial, participants were asked to imagine improvising a melody piece according to different cues after a cross was shown for 2 s. The *Familiar* condition, *Unfamiliar* condition or *Baseline* condition appeared randomly and lasted for 14 s in the paradigm (The period of 14 s was determined by professional composers. It was enough for them to read the cues and create new music in an efficient way). After each imagery improvisation, participants were asked to give an evaluation score of their performance in a box within 4 s. The evaluation results were recorded via an MRI-compatible button box. During *Baseline* conditions, participants were asked to think about nothing with no response on the button. The tasks were presented in two runs. Each run consisted of 10 trials of *Familiar* condition, 20 trials of *Unfamiliar* condition and 10 trials of *Baseline* condition, which were presented randomly (Figure 1).

#### Behavioral pilot

First, each participant performed a behavioral pilot on a computer outside the magnetic resonance imaging (MRI) scanner. Participants were instructed to follow the instructions on the screen, perform imagery improvisation and complete the evaluation by pressing the keyboard with their right hand. After the pilot, an interview was conducted to confirm familiarity with the paradigm and the ability to imagine improvisations. Thus, we ensured the eligibility of participants for MRI scanning.

#### MRI scanning

Images were acquired on a 3T magnetic resonance imaging (MRI) scanner (GE Discovery MR750, USA) at the MRI Research Center of UESTC using a standard GE whole head coil.

During scanning, we used foam padding and ear plugs to reduce head motion and scanning noise, respectively. For the group of composers, the task fMRI scanning was conducted with the same paradigm as the pilot. Importantly, participants were asked to follow the instructions on the screen and to move as little as possible when pressing a button on the keyboard. Functional images were acquired using echo-planar imaging (EPI) sequences, and the parameters of both resting-state and task scanning with an eight-channel phased array head coil were as follows: repetition time (TR) = 2,000 ms, echo time (TE) = 30 ms, flip angle (FA) = 90°, matrix = 64 × 64, field of view (FOV) = 240 × 240 mm, and slice thickness = 4 mm (with a gap of 0.4 mm). The first five volumes were discarded due to magnetization equilibrium. During the first and second functional image runs, anatomical T1-weighted images were recorded between the first acquired using a 3-dimensional fast spoiled gradient echo (T1-3D FSPGR) sequence [TR = 5.948 ms, TE = 1.964 ms, FA = 9°, matrix = 256 × 256, FOV = 204 × 163 mm, slice thickness 1 mm (no gap), 154 slices]. For the control group, only anatomical T1-weighted images were collected, with the same parameters above.

### Data analysis

#### Behavioral data analysis

The mean value and the standard deviation (SD) of the evaluation scores by each composer in an fMRI session were calculated to assess their improvisation status during scanning. Additional demographic properties such as age, years of musical improvisation training and MIL scores were also analyzed by statistical methods.

#### Functional imaging analysis

##### Preprocessing

fMRI data were preprocessed using the SPM8 software package (statistical parametric mapping, http://www.fil.ion.ucl.ac.uk/spm/). Each dataset was realigned. The time was corrected to reflect differences in image acquisition time between slices, and datasets then underwent normalization to transform images to match the template from the Montreal Neurological Institute (MNI) atlas space (Evans et al., 1993). Then, images were resampled to 3 × 3 × 3 mm^3^ and spatially smoothed with an 8-mm full-width at half-maximum (FWHM) kernel.

##### Statistical tests

We conducted the standard second-level analysis embedded in the SPM software. Three main contrasts were specified per single-participant analysis: (1) *Familiar* vs. *Baseline*, (2) *Unfamiliar* vs. *Baseline*, and (3) *Unfamiliar* vs. *Familiar*. For first level analyses, data were analyzed on a pixel level using a GLM for each subject (Herholz et al., 2016) using SPM8 software. The two regressors (*Familiar* and *Unfamiliar*) were modeled on two successive repetition times during listening and then convolved with the hemodynamic response function. The GLM also included six regressors for participant motion and a constant term. The resulting single participant contrast images were then entered into second-level random-effects group analyses for each of the corresponding contrasts to assess basic task-related activation and differences between *Unfamiliar* and *Familiar* conditions for each voxel. Following other published papers (Lu et al., 2015; He et al., 2017), the statistical threshold was set at a whole-brain false discovery rate of *P* < 0.05 for main effects combined with an extent threshold of at least 600 mm^3^ for all reported clusters.

##### Analysis based on region of interest (ROI)

To investigate the participation of the MD system in musical improvisation, we defined six 6-mm radius spherical ROIs based on Duncan's report (Duncan, 2010) and conducted one sample *t*-tests for different conditions.

##### Correlations with musical improvisational level

To study the relationship between functional imaging and levels of improvisation variables, we chose regions that showed significant changes and calculated the average value of the regression coefficients as a *z*-value. Then, we computed the correlation between the average *z*-value and the MIL scores with a partial correlation analysis, which included the covariates of age and gender (Tan et al., 2015).

##### Functional connectivity

Based on the results of abovementioned analysis of ROIs, the IPS was found have a higher activation under the *Unfamiliar* condition. To examine the role that this key component in the MD system played with other brain regions, we used the generalized form of context-dependent psychophysiological interactions (gPPI) analysis (McLaren et al., 2012; Gao et al., 2016) to compute the functional connectivity between the bilateral IPS and the whole brain.

#### Structural covariance analysis

We are curious about whether long-term training on musical improvisation can affect the structure of brain, thus we did the structural covariance analysis.

##### Measurement of cortical thickness

For both composers and controls, T1-weighted images were processed by the CIVET pipeline (version 2.0) developed at the Montreal Neurological Institute (Ad-Dab'bagh et al., 2006). In the pipeline, images were first corrected using the N3 algorithm (Sled et al., 1998) and registered to the ICBM152 space (Collins et al., 1994). Afterwards, brain volumes were classified into gray matter (GM), white matter (WM) and cerebrospinal fluid. The CLASP algorithm was used to extract the inner (GM-WM interface) and outer (pial) cortical surfaces, which consisted of 40,962 vertices in each hemisphere (Kim et al., 2005). These surfaces were nonlinearly aligned to a hemisphere-unbiased iterative surface template (Lyttelton et al., 2007). Then, we measured cortical thickness by the Euclidean distance between linked vertices on the inner and outer cortical surfaces throughout the cortex with the *tlink* metric (Lerch and Evans, 2005).

##### Analysis of structural covariance

We calculated structural covariance by correlating the cortical thickness of each seed (IPS, IFS, AI/FO, RPFC, preSMA, ACC) with the thickness of all other surface points of the entire cortex in the composer group and the control group (Suh et al., 2016).

## Results

### Behavioral results

**Figure 2:**
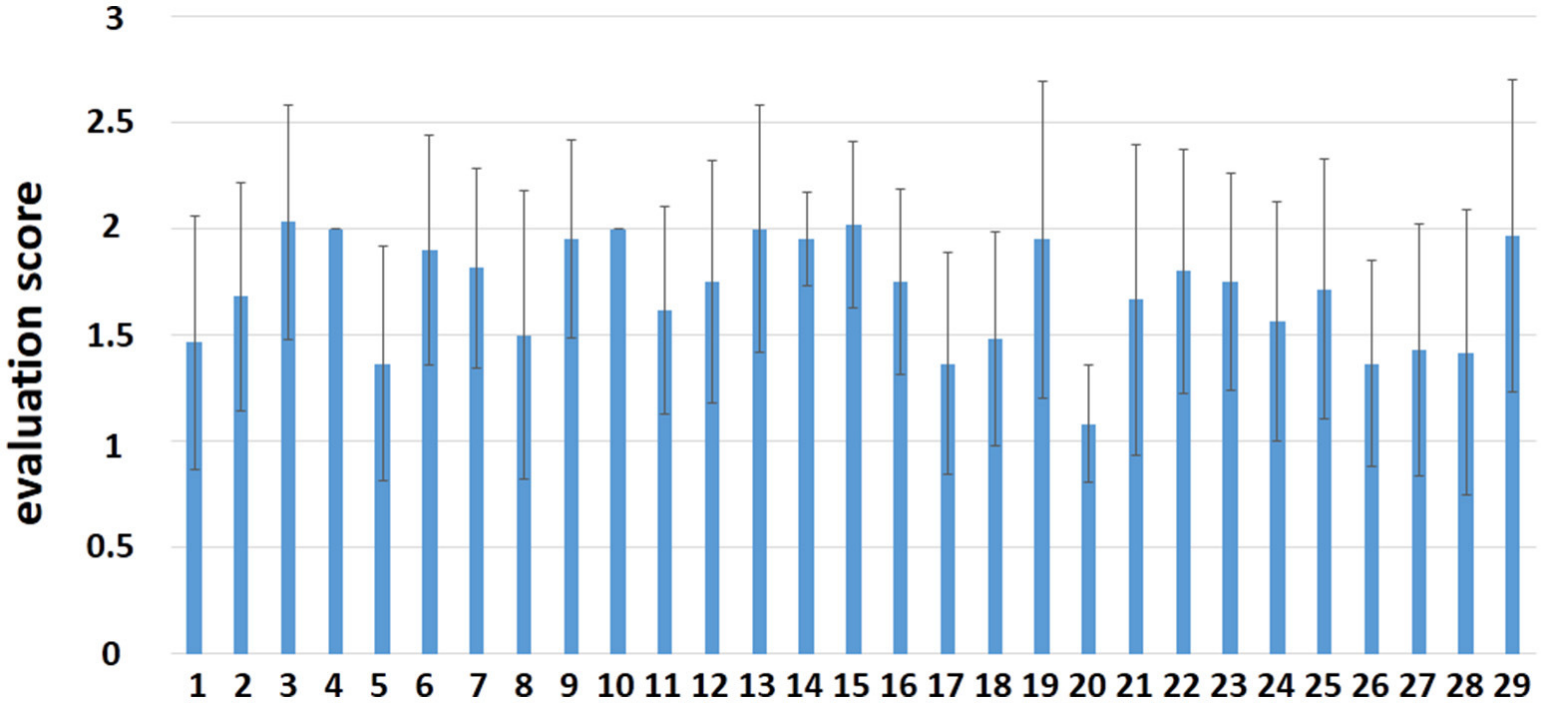
The evaluation score by each participant in the fMRI session.

The mean value and the standard deviation (SD) of the evaluation scores by each composer in the fMRI session are shown in Figure 2. From the intermediate overall scores given by participants, we can infer that participants were positively involved in the improvisation task during scanning. The results of the evaluation scores are just shown here but were not involved in subsequent analyses. The demographics of the 29 composers and 31 controls are shown in Table 1.

**Table 1:** Demographics of all participants.

	**Composers (Mean ± SD)**	**Controls (Mean ± SD)**
Age (years)	19.79 ± 1.45	20.16 ± 2.38
Gender	14 males/15 females	18 males/13 females
Years of improvisation training	3.36 ± 0.67	–
MIL score	79.37 ± 4.97	–

### Functional imaging results

#### Activation maps

We compared the activation maps across different improvisation conditions and baseline to assess basic task-related activation.

**Figure 3:**
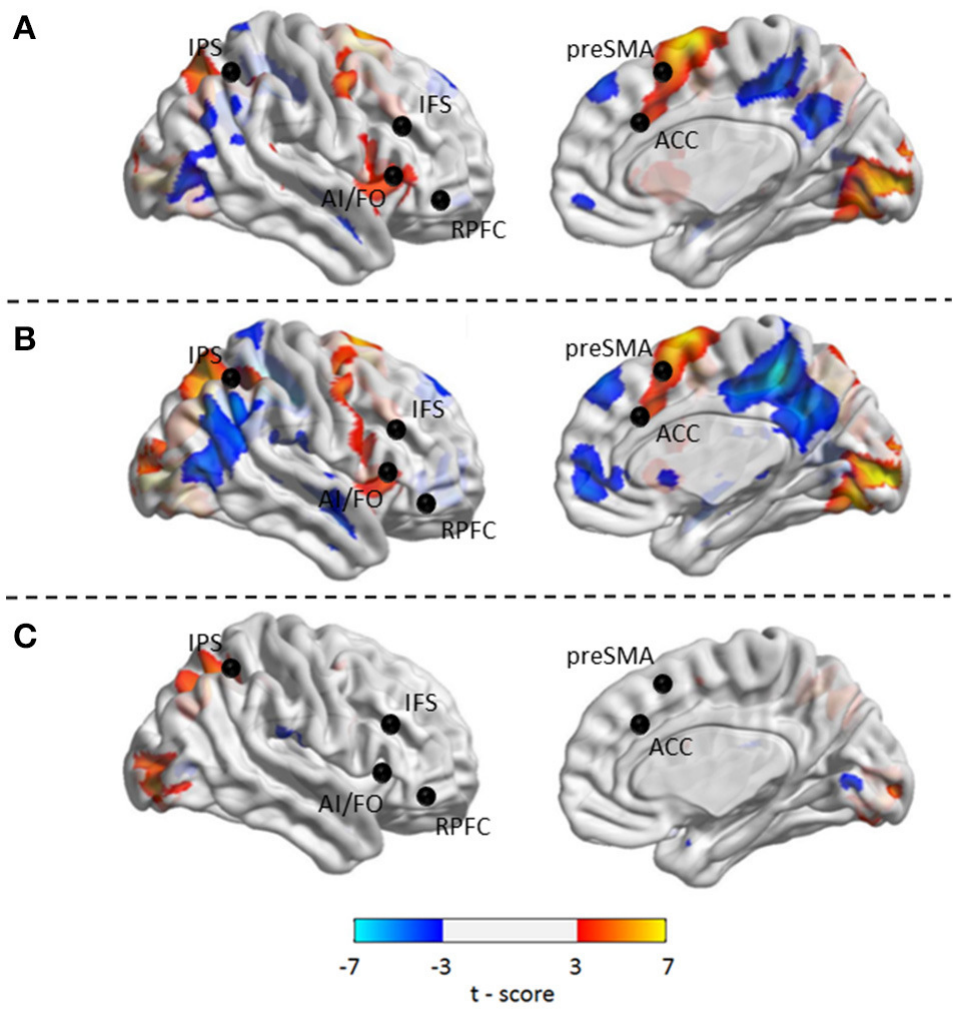
Activated regions under different contrasts. **(A)** Functional activation changes between *Familiar* and *Baseline* conditions. **(B)** Functional activation changes between *Unfamiliar* and *Baseline* conditions. **(C)** Functional activation changes between *Unfamiliar* and *Familiar* conditions. Left hemisphere peaks have been transposed to the right.

Compared with *Baseline*, the *Familiar* condition mainly activated the bilateral supplementary motor area, bilateral precentral gyrus, left postcentral gyrus, bilateral inferior parietal lobule, bilateral superior parietal lobule, bilateral inferior frontal gyrus, bilateral superior frontal gyrus, bilateral middle frontal gyrus, bilateral middle occipital gyrus, bilateral superior occipital gyrus, and bilateral superior temporal gyrus [Table 2 and Figure 3A, false discovery rate (FDR)-corrected *p* < 0.05, cluster size > 600 mm^3^]. The *Unfamiliar* condition mainly activated the bilateral supplementary motor area, bilateral precentral gyrus, left postcentral gyrus, bilateral inferior parietal lobule, bilateral superior parietal lobule, bilateral inferior frontal gyrus, bilateral superior frontal gyrus, bilateral middle frontal gyrus, bilateral middle occipital gyrus, bilateral superior occipital gyrus, and left superior temporal gyrus (Table 3 and Figure 3B, FDR-corrected *p* < 0.05, cluster size > 600 mm^3^).

**Table 2:** Activation under the *Familiar* condition.

**Region**	**Laterality**	**BA**	**MNI coordinates (mm)**			**T score**	**Cluster (Voxels)**
			**x**	**y**	**z**		
Supplementary motor area	L	6	−3	3	69	11.77	13,999
Supplementary motor area	R	6	1	3	69	8.61	
Precentral gyrus	L	6	−48	−3	51	8.54	
Precentral gyrus	R	6	54	0	48	5.45	
Postcentral gyrus	L	6	−60	0	16	4.21	
Inferior parietal lobule	L	40	−39	−45	42	5.79	
Inferior parietal lobule	R	40	48	−39	48	4.04	
Superior parietal lobule	L	7	−15	−75	51	7.72	
Superior parietal lobule	R	7	21	−70	51	5.11	
Inferior frontal gyrus	L	44	−54	9	18	6.70	
Inferior frontal gyrus	R	9	63	15	30	3.53	
Superior frontal gyrus	L	6	−21	3	65	4.71	
Superior frontal gyrus	R	6	24	0	54	4.54	
Middle frontal gyrus	L	6	−27	5	57	3.73	
Middle frontal gyrus	R	6	32	1	58	3.34	
Middle occipital gyrus	L	7	−27	−66	39	5.72	
Middle occipital gyrus	R	39	30	−63	36	5.34	
Superior occipital gyrus	L	7	−23	−74	39	4.57	
Superior occipital gyrus	R	7	26	−68	39	4.15	
Superior temporal gyrus	L	22	−52	12	−3	6.50	
Superior temporal gyrus	R	42	60	−33	12	3.45	118

*L, left; R, right; BA, Brodmann area; FDR-corrected p < 0.05, cluster size > 600 mm^3^*.

**Table 3:** Activation under the *Unfamiliar* condition.

**Region**	**Laterality**	**BA**	**MNI coordinates (mm)**			**T score**	**Cluster (Voxels)**
			**x**	**y**	**z**		
Supplementary motor area	L	6	−3	3	69	9.65	17,337
Supplementary motor area	R	6	2	3	69	7.22	
Precentral gyrus	L	6	−48	0	54	8.89	
Precentral gyrus	R	6	57	6	45	5.50	
Postcentral gyrus	L	6	−56	−1	41	5.71	
Inferior parietal lobule	L	40	−39	−45	42	7.17	
Inferior parietal lobule	R	40	36	−49	42	4.08	
Superior parietal lobule	L	7	−21	−72	48	7.50	
Superior parietal lobule	R	7	22	−68	55	6.32	
Inferior frontal gyrus	L	44	−51	9	18	7.63	
Inferior frontal gyrus	R	44	50	12	18	3.65	
Superior frontal gyrus	L	6	−24	−2	65	6.04	
Superior frontal gyrus	R	6	22	6	55	3.80	
Middle frontal gyrus	L	6	−30	4	55	4.89	
Middle frontal gyrus	R	6	33	3	58	4.40	
Middle occipital gyrus	L	19	−30	−71	40	4.95	
Middle occipital gyrus	R	19	33	−73	40	5.04	
Superior occipital gyrus	L	7	−18	−76	41	5.48	
Superior occipital gyrus	R	7	26	−73	41	5.59	
Superior temporal gyrus	L	22	−53	13	−8	5.15	

*L, left; R, right; BA, Brodmann area; FDR-corrected p < 0.05, cluster size > 600 mm^3^*.

Afterwards, the contrast between *Unfamiliar* and *Familiar* conditions was examined. Under the *Unfamiliar* condition, a stronger activation appeared in the left precentral gyrus, left inferior frontal gyrus, and bilateral inferior parietal lobule. Additionally, the bilateral inferior occipital gyrus, bilateral middle occipital gyrus, and bilateral superior occipital gyrus also appeared to have stronger activation. Under the *Familiar* condition, higher activation was found in the temporal regions such as the right superior temporal gyrus (Table 4 and Figure 3C, FDR-corrected *p* < 0.05, cluster size > 600 mm^3^).

**Table 4:** Contrast between *Unfamiliar* and *Familiar* conditions.

**Region**	**Laterality**	**BA**	**MNI coordinates (mm)**			**T score**	**Cluster (Voxels)**
			**x**	**y**	**z**		
Precentral gyrus	L	9	−54	10	36	4.43	142
Inferior parietal lobule	L	40	−42	−39	45	3.70	23
Inferior parietal lobule	R	40	32	−52	44	3.77	
Superior parietal lobule	L	7	−23	−62	44	3.72	
Superior parietal lobule	R	7	26	−65	51	4.67	
Inferior frontal gyrus	L	9	−57	12	27	3.55	
Superior frontal gyrus	L	6	−23	−3	53	4.28	96
Inferior occipital gyrus	L	18	−33	−84	−4	4.49	
Inferior occipital gyrus	R	18	32	−86	−3	5.97	
Middle occipital gyrus	L	18	−35	−87	−3	5.38	
Middle occipital gyrus	R	19	31	−86	4	4.50	
Superior occipital gyrus	L	17	−14	−92	3	5.06	
Superior occipital gyrus	R	7	27	−67	42	5.35	
Superior temporal gyrus	R	42	57	−29	17	−3.97	41

*L, left; R, right; BA, Brodmann area; FDR-corrected p < 0.05, cluster size > 600 mm^3^*.

#### Analysis based on ROIs

**Figure 4:**
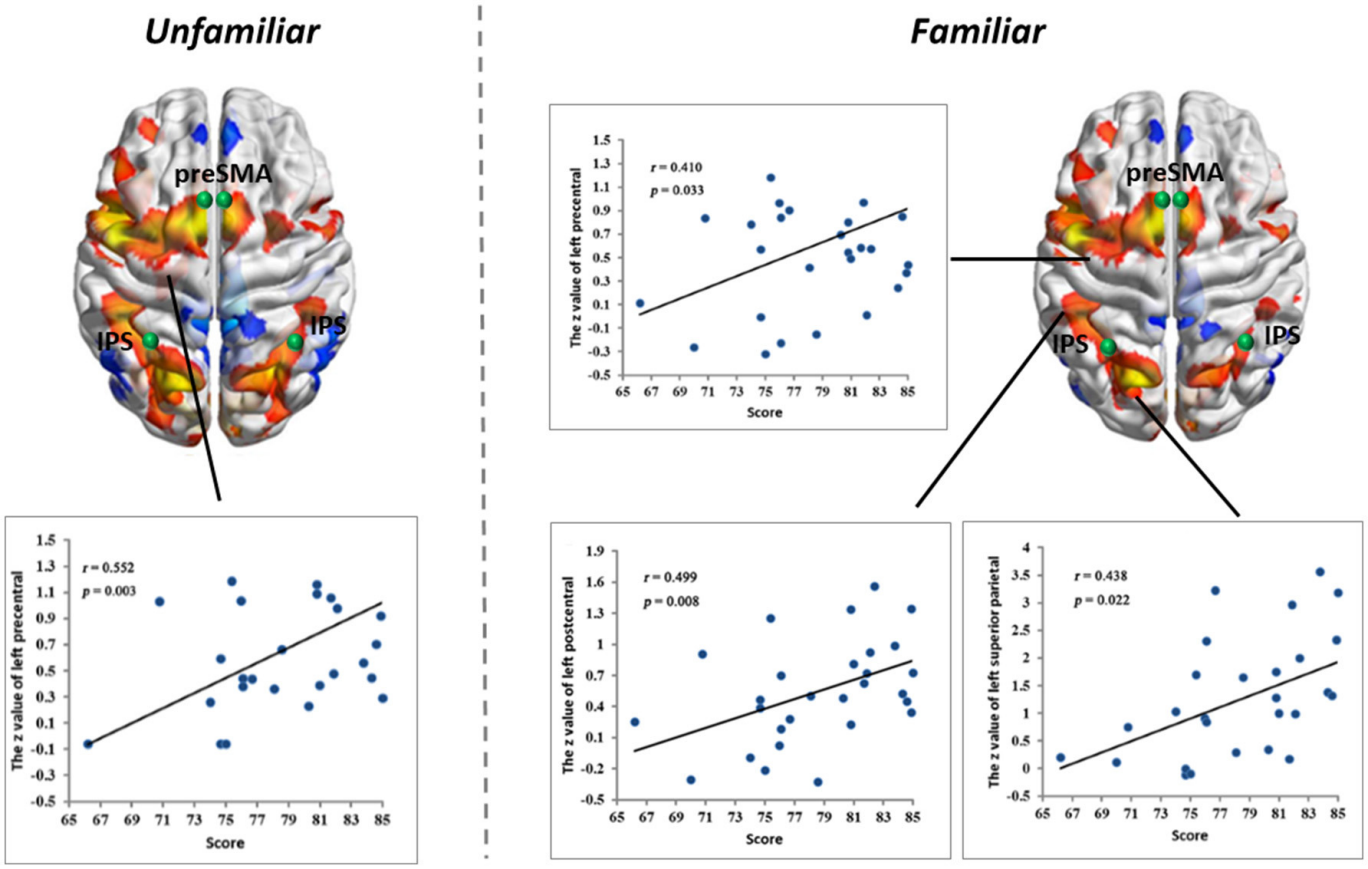
Linear partial correlation coefficients (*r*) between the average *z* values of ROIs in the MD system and MIL scores. **(Left)** Linear partial correlation in the left precentral gyrus under the *Familiar* condition (*p* < 0.05). **(Right)** Linear partial correlation in the left precentral gyrus, left postcentral gyrus and left superior parietal lobule under the *Familiar* condition (*p* < 0.05).

We compared six ROIs between the different conditions using one sample *t*-tests. The results are shown in Table 5 and Figure 4. Thus, we can elucidate that most of the areas within the MD system were involved in musical improvisation. However, MD system activation did not show any differences when the *Unfamiliar* condition was compared with *Familiar* condition.

**Table 5:** The comparisons of six ROIs between different conditions (FDR-corrected, *p* < 0.05).

	**IPS**	**IFS**	**AI/FO**	**preSMA**	**RPFC**	**ACC**
Unfamiliar-baseline	***t*** = **4.19**	***t*** = **3.83**	***t*** = **4.59**	***t*** = **4.40**	*t* = −0.96	*t* = 0.81
	***p*** = **0.0002**	***p*** = **0.0007**	***p*** = **0.0001**	***p*** = **0.0001**	*p* = 0.35	*p* = 0.43
Familiar-baseline	***t*** = **3.41**	***t*** = **3.44**	***t*** = **4.78**	***t*** = **4.46**	*t* = −0.18	*t* = 1.48
	***p*** = **0.002**	***p*** = **0.002**	***p*** = **0.0001**	***p*** = **0.0001**	*p* = 0.86	*p* = 0.15
Unfamiliar-familiar	***t*** = **2.19**	*t* = 0.31	*t* = −0.93	*t* = 0.14	***t*** = −**3.46**	*t* = −1.74
	***p*** = **0.04**	*p* = 0.76	*p* = 0.36	*p* = 0.89	***p*** = **0.001**	*p* = 0.09

*The ROIs were chosen based on Duncan's report (Duncan, 2010), which included IFS (41, 23, 29), AI/FO (35, 18, 2), preSMA (0, 18, 50), ACC (0, 31, 24), IPS (37, −56, 41) and RPFC (21, 43, −10). Coordinates are shown in MNI space. The bold values indicate the significant differences*.

#### Correlations with musical improvisational level

We calculated linear partial correlation coefficients between the average *z*-values and MIL scores. Significant correlations were found between adjacent areas of the MD system and MIL scores in both the *Familiar* and *Unfamiliar* conditions.

#### Functional connectivity

**Figure 5:**
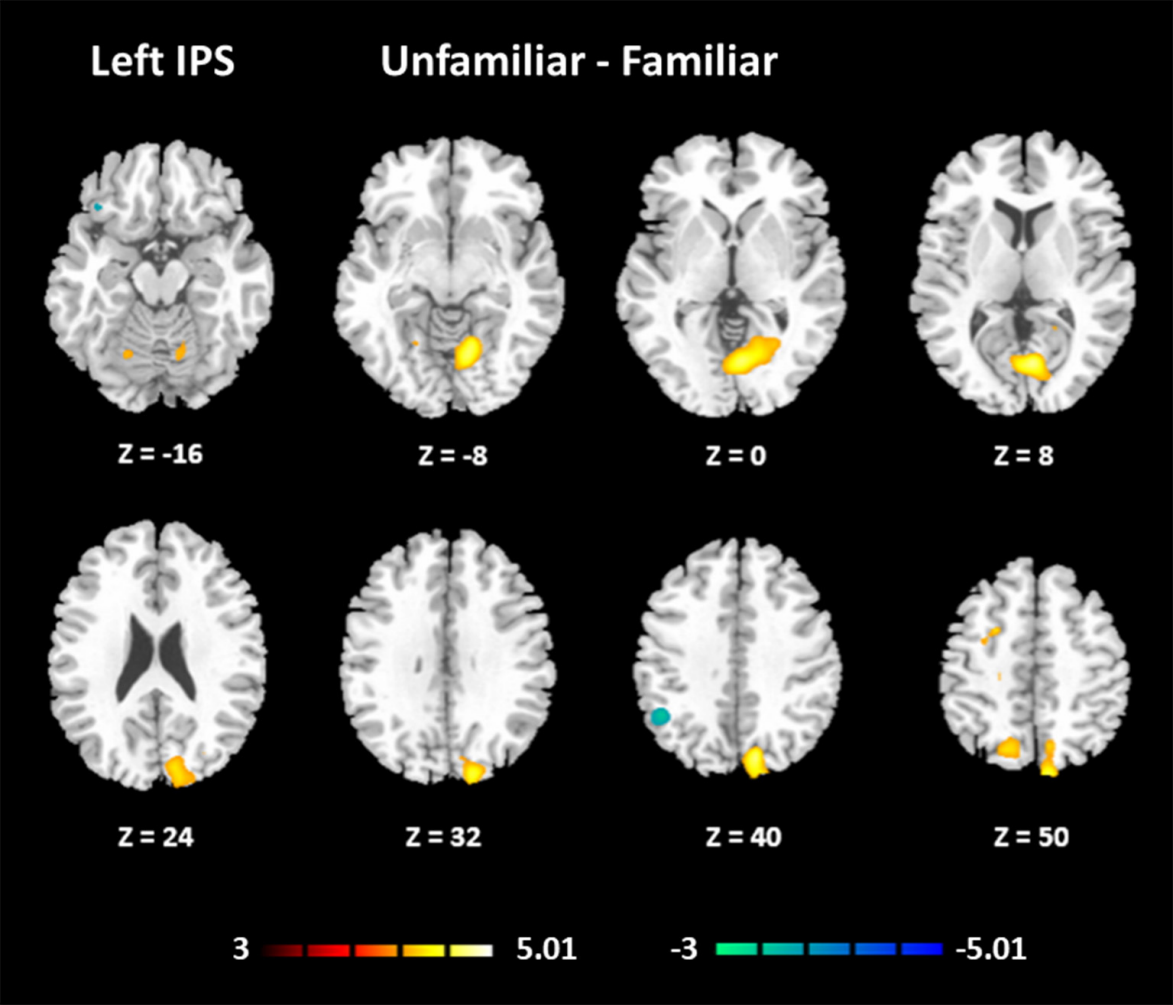
Functional connectivity based on the seed of the left IPS (FDR-corrected *p* < 0.05).

**Figure 6:**
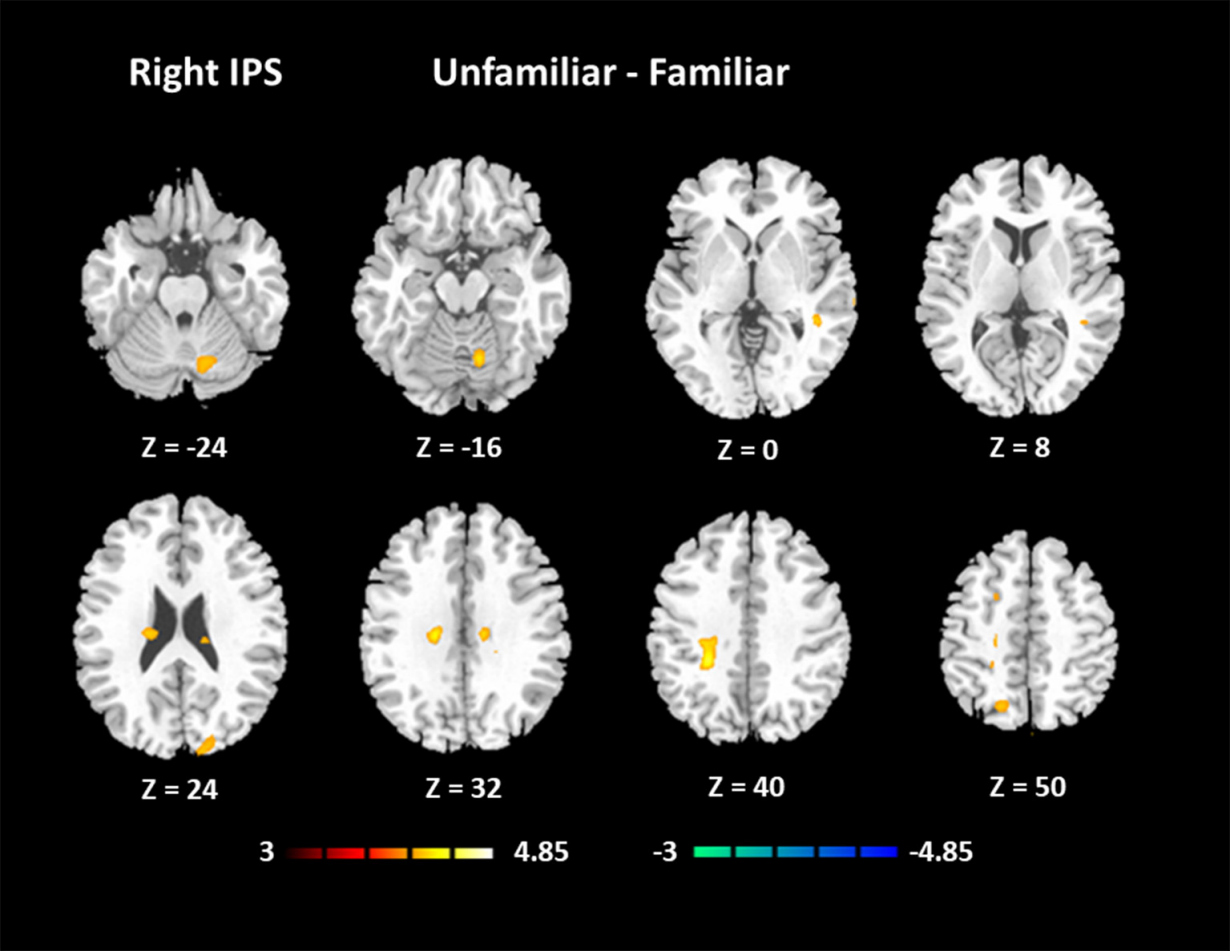
Functional connectivity based on the seed of the right IPS (FDR-corrected *p* < 0.05).

The results for the functional connectivity between the left IPS and the whole brain are shown in Table 6 and Figure 5. The results for the functional connectivity between the right IPS and the whole brain are shown in Table 7 and Figure 6. From these results, we could see that visual regions such as the middle occipital gyrus and superior occipital gyrus had the main functional connectivity with the bilateral IPS.

**Table 6:** Results of functional connectivity assessments based on the seed of the left IPS.

**Region**	**Laterality**	**BA**	**MNI coordinates (mm)**			**T score**	**Cluster (Voxels)**
			**x**	**y**	**z**		
Lingual_L/Occipital_Sup_L	L	19	−9	−87	45	5.01	3,788
Occipital_Mid_R	R	19	36	−78	18	2.67	61
Supp_Motor_Area_L	L	6	−6	0	66	2.63	34
Angular_R	R	39	48	−54	36	−3.96	301
Frontal_Sup_Medial_R	R		6	51	51	−3.16	199
Temporal_Inf_R	R	20	51	12	−36	−3.49	133
Frontal_Mid_R	R		42	9	60	−3.10	107
Frontal_Inf_Tri_L	L	44	−57	15	18	−3.27	96
Temporal_Inf_L	L	20	−42	−3	−33	−3.66	94
Frontal_Inf_Orb_R	R	38	42	27	−24	−3.44	74

**Table 7:** Results of the functional connectivity assessments based on the seed of the right IPS.

**Region**	**Laterality**	**BA**	**MNI coordinates (mm)**			**T score**	**Cluster (Voxels)**
			**x**	**y**	**z**		
Occipital_Sup_L/Lingual_L	L	18	−12	−66	−15	3.66	2,327
Precuneus_R/Parietal_Sup_R	R		27	−39	42	4.85	1,048
Temporal_Mid_L	L	21	−45	−42	0	3.82	448
Putamen_R	R		30	−9	−3	2.93	250
Putamen_L	L		−18	9	3	2.93	225
Frontal_Mid_L	L	45	−48	45	15	3.46	107
Temporal_Pole_Sup_R	R	21	63	6	−9	3.10	105
Frontal_Inf_Orb_L	L	47	−30	36	−9	2.81	56
Supp_Motor_Area_L	L	6	−3	0	69	2.75	29

#### Structural covariance results

**Figure 7:**
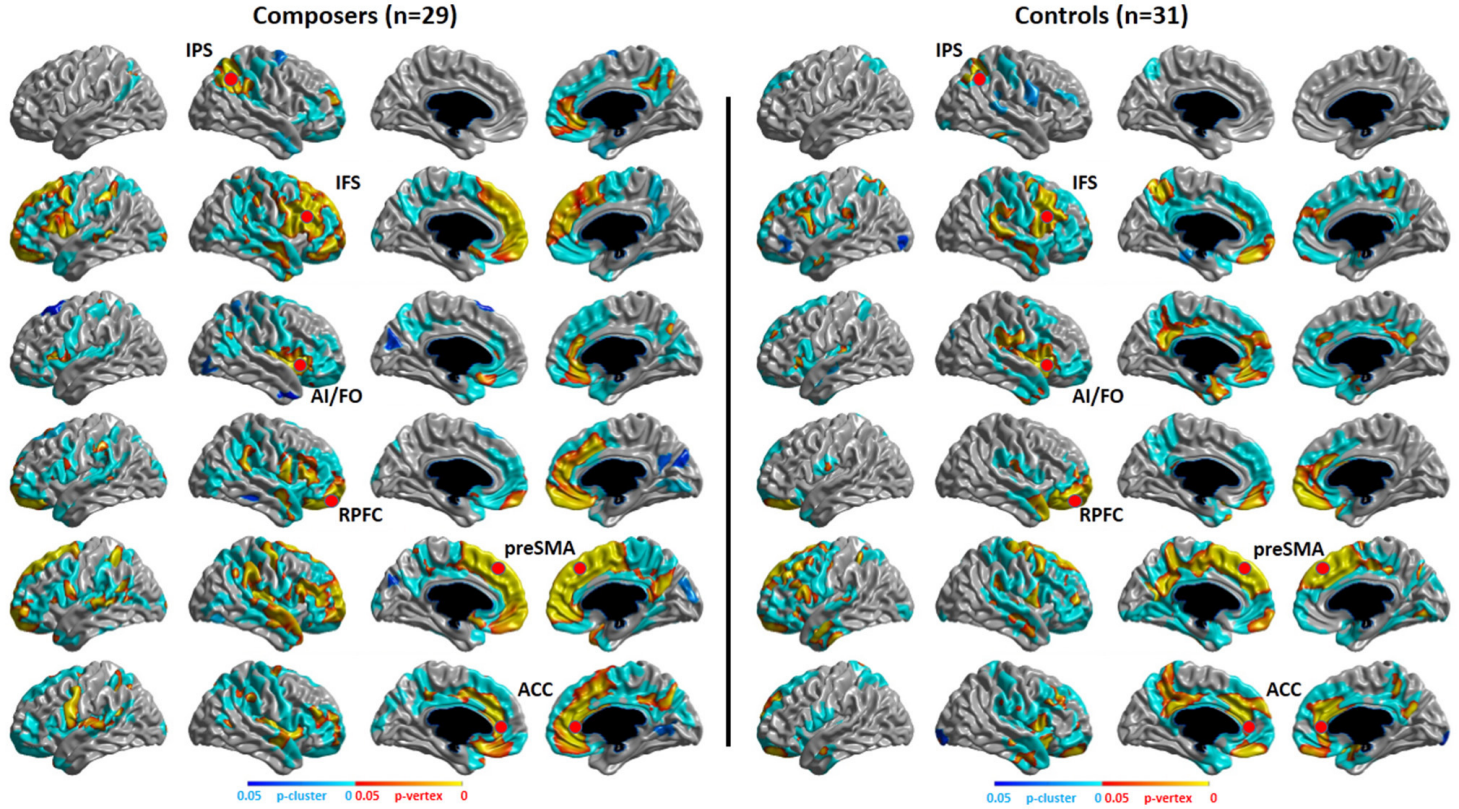
*P*-map of structural covariance. Significant correlations of cortical thickness with the seed points of the MD system (showed in red points) in composers and controls are mapped, *p* < 0.05, RFT (Random Field Theory)-corrected.

The structural covariance results of the composer group and the control group are shown in Figure 7. We can conclude from the results that the composer group has a stronger distribution of structural covariance than the control group.

## Discussion

### The MD system during musical improvisation

Because improvisation with familiar cues involves more working memories of music, and unfamiliar cues involve more creative novelty, we used these stimuli to examine the specific brain regions involved in each condition of musical improvisation. The results show that the involvement of the MD system can be found during improvisation with both familiar and unfamiliar cues. Combined with previous findings, we can infer that the main role of the MD system is dealing with the novelty of a task while participating in working memory and attentional control during musical improvisation.

First, musical improvisation needs the creative competency of novelty (Gross and Seashore, 1941), for example, a germinal idea (Bennett, 1976). In line with previous studies (Berkowitz and Ansari, 2008; Limb and Braun, 2008), the results of the current study showed that the prefrontal regions of the MD system are included in musical improvisation activities, which are related to the invention of novel motor sequences. More specifically, the dorsal premotor cortex within these areas plays a key role in abstract auditory-motor mapping, due to the imagining of novel music required by our tasks (Hoshi and Tanji, 2006; Zatorre et al., 2007). Additionally, the greater activation shown in the left premotor area implies a higher involvement of complex demands when improvising novel melodies (Haaland et al., 2004). Importantly, our results extend previous findings that musical improvisation is associated with parietal regions. In general, parietal areas play important roles in visuomotor control and the selection and spatial execution of movements (Wise et al., 1997; Bengtsson et al., 2007). Studies show that the superior parietal cortex is involved in auditory-motor transformations of musical structure (Brown et al., 2013) and spatial notations to motor responses (Stewart et al., 2003). One possible explanation is that the hierarchy of novel musical structure specifically relies on the parietal lobe. In addition, this finding of parietal activation is consistent with the findings of experiments on mental imagery (Herholz et al., 2012).

Second, the activation of the MD system in both conditions could also be explained by the function of working memory during improvisation. Improvisation needs a rich musical background including musical appreciation, knowledge of theory, and performance experience (Gross and Seashore, 1941). Thus, during the first stage of improvisation, an entire melody piece must be organized by selecting from musical materials in working memory. After this stage, the repetitive elements would be initiated under the *Familiar* condition and inhibited under the *Unfamiliar* condition (Nathaniel-James and Frith, 2002).

Responses given by the participants illustrated that they attentively improvised music; thus, the involvement of the MD system could also be interpreted by the requirements of attention. Activation of the dorsolateral superior frontal gyrus, which is part of the MD system, is involved in attentional control (Desimone and Duncan, 1995). One possible explanation is that musical improvisation demands a top-down control of attention to musical materials, especially a higher demand under the condition of improvising new melodies. Moreover, attentively processing music is found to involve other regions including the premotor and parietal cortex as a function of recruiting neural circuits of working memory and motor imagery (Janata et al., 2002).

In addition, we noticed that the IPS within the MD system had a higher activation under the *Unfamiliar* condition. The IPS has been reported to participate in musical transposition (Foster and Zatorre, 2010; Foster et al., 2013), where a melody is shifted to a new pitch height but still maintains its pitch interval structure. Additionally, musical training can improve the ability to utilize relative pitch information, especially for unfamiliar melodies (Dowling and Harwood, 1986). Thus, the greater recruitment of the IPS would lead us to infer that improvising unfamiliar melodies could rely on more strategies, such as musical transposition manipulation. The further analysis of functional connectivity showed that visual regions had the main functional connectivity with bilateral IPS, especially under the *Unfamiliar* condition. This finding could be interpreted that tonal/atonal rehearsal based on working memory processing for visual stimuli was much more strongly involved in improvising with unfamiliar cues (Schulze et al., 2011), and details need to be confirmed by further studies.

Finally, the influence of the experimental tasks should also be addressed. In our study, we used mental imagery tasks to simulate improvisational activities due to the lack of an MRI-compatible keyboard. However, previous studies have shown the involvement of some parts of the MD system (such as the SMA and IPS) during imagery tasks (Herholz et al., 2012). Are these parts activated by the imagery task or by musical improvisation? We found some reports that the SMA and IPS are included in a fronto-parietal network that could be involved in both motor execution and motor imagery (Meister et al., 2004). Thus, we can infer that the main activation of those parts was caused by musical improvisation. Nevertheless, further experiments still need to be completed to confirm this issue.

### Evidence for neuroplasticity

Our functional results showed that some areas within the MD system were positively correlated with the level of musical improvisation. These areas included the left precentral gyrus under the *Familiar* condition and the left postcentral gyrus, left precentral gyrus, and left superior parietal regions under the *Unfamiliar* condition. The finding of a predominant correlation between the left areas of the brain and MIL scores implies a higher involvement of the aforementioned complex demands (Haaland et al., 2004), as well as the habit of writing (Menon and Desmond, 2001), during musical improvisation training. Moreover, the structural data also provided evidence that the composer group had a stronger distribution of structural covariance than the control group. Therefore, similar to data on the functional changes in the brains of other experts (Duan et al., 2012; Gong et al., 2015; Li et al., 2015), our results also supported the idea that neuroplasticity can be affected by domain-specific training.

### The auditory cortex during musical improvisation

The auditory cortex showed activation in all conditions and a significantly stronger activation when improvising with familiar cues. First, these results support the notion that auditory regions can be activated with music-related tasks via auditory-motor interactions regardless of auditory stimuli (Zatorre et al., 2007). Second, stronger activation appeared with familiar cues, which required more memory resources, probably because some regions, such as the superior temporal cortex, are involved in auditory working memory processing (Hickok and Poeppel, 2000; Gaab et al., 2003).

To our knowledge, auditory cortices can be activated whether or not there are real motor activities when referring to musical activities (Halpern and Zatorre, 1999; Janata, 2001). These findings can lead to a discussion about the differences between improvisation without motor execution and mentally rehearsing a melody. However, studies have shown that motor-functional parts of the MD system, such as the SMA, could be activated during auditory imagery tasks (Zatorre et al., 1996; Rao et al., 1997). Considering that imagery tasks involve complex processing, the relationship between auditory cortices and the MD system based on different task designs remains to be determined.

## Conclusion

In this study, we provided evidence that the MD system strongly participated in musical improvisation. Our results suggested that musical improvisation was an activity with complex demands in which the MD system mainly contributed to the novelty of melodies, working memory, and attentional control. In particular, the higher IPS recruitment indicated that musical transposition manipulation was highly involved in improvising unfamiliar melodies. Both functional and structural analyses indicated evidence of neuroplasticity in MD regions that could be associated with musical improvisation training. These findings can help unveil the functional mechanisms of the MD system in musical cognition, as well as improve our understanding musical improvisation.

Nevertheless, this study still has some limitations that should be addressed here. Firstly, although strong evidence has been found that the MD system is involved in musical improvisation, a longitudinal study is still needed in the future. Secondly, in order to do a better manipulation check, an MRI-compatible keyboard for recording realtime music is indispensable in the following study. Besides, since we infer that MD system participated in musical improvisation through processing the novelty of melodies, working memory, and attention, it is also necessary to inspect how these functions interact with each other within the MD system. Moreover, improvisation is highly dependent on musical genre, which means that differences between improvising in a classical style and improvising in a jazz style should also be investigated. At last, investigations on how the auditory cortex relates to the MD system should be completed as well, as this could help find other cognitive components of musical improvisation.

## Author contributions

JL, HY, AE, and DY: Substantial contributions to the conception or design of the work; JL, HY, and CH: Data acquisition; JL, HH, and SJ: Data analysis; JL, and DY: Drafting the work and revising it critically for important intellectual content; JL, AE, and DY: Final approval of the version to be published.

### Conflict of interest statement

The authors declare that the research was conducted in the absence of any commercial or financial relationships that could be construed as a potential conflict of interest.
